# MSGNN-DTA: Multi-Scale Topological Feature Fusion Based on Graph Neural Networks for Drug–Target Binding Affinity Prediction

**DOI:** 10.3390/ijms24098326

**Published:** 2023-05-05

**Authors:** Shudong Wang, Xuanmo Song, Yuanyuan Zhang, Kuijie Zhang, Yingye Liu, Chuanru Ren, Shanchen Pang

**Affiliations:** 1 Qingdao Institute of Software, College of Computer Science and Technology, China University of Petroleum, Qingdao 266580, China; 2 School of Information and Control Engineering, Qingdao University of Technology, Qingdao 266525, China

**Keywords:** drug–target binding affinity prediction, graph neural networks, feature representation learning

## Abstract

The accurate prediction of drug–target binding affinity (DTA) is an essential step in drug discovery and drug repositioning. Although deep learning methods have been widely adopted for DTA prediction, the complexity of extracting drug and target protein features hampers the accuracy of these predictions. In this study, we propose a novel model for DTA prediction named MSGNN-DTA, which leverages a fused multi-scale topological feature approach based on graph neural networks (GNNs). To address the challenge of accurately extracting drug and target protein features, we introduce a gated skip-connection mechanism during the feature learning process to fuse multi-scale topological features, resulting in information-rich representations of drugs and proteins. Our approach constructs drug atom graphs, motif graphs, and weighted protein graphs to fully extract topological information and provide a comprehensive understanding of underlying molecular interactions from multiple perspectives. Experimental results on two benchmark datasets demonstrate that MSGNN-DTA outperforms the state-of-the-art models in all evaluation metrics, showcasing the effectiveness of the proposed approach. Moreover, the study conducts a case study based on already FDA-approved drugs in the DrugBank dataset to highlight the potential of the MSGNN-DTA framework in identifying drug candidates for specific targets, which could accelerate the process of virtual screening and drug repositioning.

## 1. Introduction

Drug discovery is a complex and time-consuming process that may span more than a decade and cost billions of dollars from screening to market [1]. Contrarily, drug repositioning provides a promising approach to overcoming the temporal and financial bottlenecks of new drug discovery. This strategy involves identifying FDA-approved drugs that exhibit a binding affinity for specific targets, alter the expression of abnormal proteins, and exert pharmacological effects [2,3,4]. The accurate identification of potential drug–target interactions is crucial for successful drug repositioning [5], and the strength of drug–target binding affinity (DTA) serves as an important indicator for drug screening [6,7,8,9,10]. Traditional methods of measuring DTA are resource-intensive and time-consuming. The rapid advancement of computer technology has facilitated accurate and efficient prediction of DTA, thereby assisting biological experiments [11]. Currently, DTA prediction methods can be classified into three categories: structure-based methods, machine learning-based methods, and deep learning-based methods.

In computer experiments, structure-based approaches typically utilize molecular docking and molecular dynamics simulations for DTA prediction. Molecular docking explores potential binding sites by considering the 3D structure of the receptor and ligand, and a scoring function based on the molecular position is defined to calculate the binding energy [12]. For proteins with known structural information, binding affinity can be obtained directly by docking the drug molecule [13]. However, this approach necessitates high-precision 3D protein structures, which may be unattainable for a massive number of proteins with unknown structural information. Even with extensive homology modelling, reliable structural information may not be acquired [14].

Conventional machine learning methods have been applied for DTA prediction. Pahikkala et al. [15] proposed KronRLS, an approach based on Kronecker regularized least squares, which utilizes the Smith–Waterman algorithm and PubChem structural clustering server to build similarity matrices for proteins and drugs, and then calculate the Kronecker product to predict the DTA. He et al. [16] introduced Simboost, which utilizes a gradient booster to extract features from drugs, targets, and drug–target pairs. These methods have limitations in achieving significant performance improvement, as they heavily rely on intricate feature engineering, which typically requires a high level of domain expertise [17].

Deep learning is widely used in many research areas of bioinformatics [18], various deep learning-based methods have been applied in DTA prediction, where it can capture complex hidden information from massive data. Öztürk et al. [9] proposed DeepDTA, which employs two convolutional neural networks (CNNs) to extract local sequence information and then feed it into several fully connected layers for DTA prediction. Similarly, Öztürk et al. [19] proposed another CNN-based model, called WideDTA, which takes advantage of two additional text-based information sources, namely protein interaction domains and ligand maximum common structure words, obtaining four representations that further improve the DTA prediction performance. Furthermore, attention-based methods have been introduced to improve interpretability in DTA prediction. Chen et al. [20] proposed TransformerCPI, which retains the decoder of the transformer but modifies its encoder and the final linear layer to increase interpretability. In another work, Yang et al. [21] developed ML-DTI, which uses a mutual information mechanism to capture the interaction relationship between drug and protein encoders, bridging the gap between the two encoders and enabling the identification of new drug–target interactions.

Although CNN-based methods have demonstrated remarkable achievements in DTA prediction, their exclusive utilization of 1D representations of drugs and proteins fails to capture the spatial structural information of molecules, such as the distance and angle between residues that determine molecular function. Graph neural networks (GNNs), renowned for their efficacy in tackling an array of challenges, have been implemented in various models representing drugs as graphs for DTA prediction. Nguyen et al. [10] devised GraphDTA, a graph-based model encoding drugs as undirected graphs represented by a feature matrix and an adjacent matrix. Experimental results and theoretical analyses suggest that graph-based drug representations may further bolster performance. Lin et al. [22] proposed DeepGS, leveraging advanced embedding learning techniques that consider molecular topology, SMILES string, and protein sequence for DTA prediction. Yang et al. [23] developed MGraphDTA, constructing a 27-layer ultra-deep GNN that learns multi-scale features and capitalizes on topological information while avoiding gradient disappearance. Furthermore, Jiang et al. [24] created DGraphDTA, which predicts the contact map using amino acid sequence, thereby constructing the protein graph to boost prediction performance further. When producing the protein contact map, WGNN-DTA [25] obviates the need for sophisticated processes such as multiple sequence alignment (MSA), which effectively enhances the execution speed.

In general, graph-based DTA prediction models only employ a limited number of GNN layers, typically ranging from two to four layers. However, such shallow GNNs are incapable of capturing the intricate topological information of molecules, leading to insufficient representation learning. To fully capture topological information, multiple layers of GNN should be stacked, which is also infeasible due to the problems of vanishing gradients and node feature degradation [26]. Furthermore, motifs have special meanings in drug molecules, such as carbon rings and NO2 groups that are prone to mutagenesis [27]. The motifs can exert their practical value when considered as a whole, and it would be meaningless if the chemical bonds in the ring are isolated separately. Therefore, motifs deserve more attention during the feature extraction process. Additionally, topological features at different scales are extracted from various GNN layers, but previous models only used single-scale features for DTA prediction, resulting in a loss of prediction performance. Therefore, the fusion of multi-scale features is necessary, and the model should be capable of adaptively fusing essential features to improve prediction performance.

In response to the aforementioned challenge, we present a novel GNN-based model for DTA prediction that leverages multi-scale topological feature fusion. Our proposed approach underscores the significance of motifs by creating drug motif-level graphs, where motifs are viewed as holistic entities and mapped as graph vertices. This design choice captures the practical value of motifs and effectively extracts meaningful features that contribute to accurate predictions. To further exploit the topological information of drug molecules, we introduce a gated skip-connection mechanism during the GNN-based representation learning process. This mechanism enables the model to dynamically adapt and selectively fuse features from different scales, thus avoiding the problems of node gradient vanishing and feature degradation. The learned enhanced representations are informative and enable accurate predictions. The experimental evaluations demonstrate that our model outperforms existing models on benchmark datasets, with low prediction error and high stability. We believe that our approach has practical applications in drug discovery and development by providing a more comprehensive and interpretable approach to DTA prediction.

This paper’s significant contributions are summarized as follows:

- To make full use of the topological information of drugs and proteins, we simultaneously construct drug atom graphs, motif graphs, and weighted protein graphs to learn drug and protein representations from multiple perspectives.
- To extract and fuse multi-scale topological information, a gated skip-connection mechanism is introduced in the feature learning based on GNNs, and topological features at different scales are selectively preserved.
- To improve the adaptive capability of the model, we incorporate an attention mechanism in the prediction phase, which enables the model to concentrate on the crucial features of multi-scale and further strengthen the DTA prediction performance.

## 2. Results

### 2.1. Evaluation Metrics

DTA prediction is a regression task using the mean squared error (MSE) as a loss function. MSE measures the error between the ground and predicted values, with a smaller MSE indicating that the predicted value is closer to the true value. MSE is defined as follows:

(1) $$\mathrm{MSE}=\frac{1}{N}\sum_{i=1}^{N}{\left(y_{i}-p_{i}\right)}^{2}$$

where $y_{i}$ is the true value of the *i*th sample and $p_{i}$ is the predicted value of the *i*th sample.

Another evaluation metric is the concordance index (CI), which measures whether the predicted values of two randomly selected drug–target pairs have a consistent relative order with the true dataset. A larger CI indicates better model prediction performance. It is defined as shown in Equation (2).

(2) $$\mathrm{CI}=\frac{1}{z}\sum_{y_{i}>y_{j}}\;h\left(p_{i}-p_{j}\right)$$

where $p_{i}$ is the prediction value for the larger affinity $y_{i}$, $p_{j}$ is the prediction value for the smaller affinity $y_{j}$, and h(x) is step function. *Z* is the normalization constant that maps the values to [0,1]. The step function is defined as shown in Equation (3).

(3) $$h(x)=\left\{\begin{array}{ccc} 0 & \;\mathrm{if}\; & x<0\\ 0.5 & \;\mathrm{if}\; & x=0\\ 1 & \;\mathrm{if}\; & x>0 \end{array}\right.$$

The Pearson correlation coefficient was calculated by Equation (4). cov(p,y) is the covariance between the predicted value *p* and true value *y*, and σ(.) is the standard deviation. A higher Pearson coefficient suggests greater predictive accuracy.

(4) $$\;\mathrm{Pearson}\;=\frac{\mathrm{cov}(p,y)}{\sigma (p)\sigma (y)}$$

Regression toward the mean ($r_{m}^{2}$) is a metric for evaluating the external predictive performance of a model. If a variable is extremely large or extremely small at this measurement, $r_{m}^{2}$ indicates how close to the mean it tends to be at the next measurement. The index calculation process is depicted in Equation (5).

(5) $$r_{m}^{2}=r^{2}\times \left(1-\sqrt{r^{2}-r_{0}^{2}}\right)$$

where *r* is the correlation coefficient with intercept and $r_{0}$ is the correlation coefficient without intercept.

### 2.2. Experimental Setup

MSGNN-DTA is built with PyTorch [28], which is an open-source machine learning framework. The GNN models are implemented using PyTorch Geometric (PyG) [29]. We evaluated the performance of the proposed model on two benchmark datasets, the Davis [30] and KIBA datasets [31]. To ensure a fair comparison, we adopted the same strategy for data partitioning as DeepDTA [9], which randomly divided the datasets into six equal parts, with one part reserved for independent testing and the remaining five parts used for model training.The hyperparameter settings for our experimental part are shown in Table 1.

### 2.3. Performance Comparison with Benchmark Models

To evaluate the superiority of MSGNN-DTA, we compared it with the state-of-the-art models on two benchmark datasets, Davis and KIBA, respectively. We compared MSGNN-DTA with KronRLS [15], SimBoost [16], DeepDTA [9], WideDTA [19], GraphDTA [10], MGraphDTA [23], GEFA [32], WGNN-DTA [25], and DGraphDTA [24], which are currently widely used benchmark methods for DTA prediction. To ensure a fair comparison, we adopted the same training and testing sets as well as performance metrics for evaluation. The performance results, along with those reported in the original publications for the baseline methods, are summarized in Table 2 and Table 3.

According to the experimental results, the proposed MSGNN-DTA achieved the best performance compared to state-of-the-art methods in all datasets, demonstrating its generalization and robustness. The model decreased the MSE by 3.5% and 7.1% and increased the CI by 0.2% and 0.4% on the Davis and KIBA datasets, respectively, underscoring the model’s ability to outperform other models in terms of predictive accuracy and reliability. Additionally, the model showed advantages in the other two evaluation metrics, $r_{m}^{2}$ and Pearson. The considerable improvements over the second best model of 1.3% and 0.5% in the Davis dataset and 2.1% and 0.8% in the KIBA dataset, respectively. Overall, the MSGNN-DTA model’s superior performance across all metrics and datasets makes it an essential tool for researchers seeking to predict drug–target affinity values.

Our proposed model achieves significant performance gains, which can be attributed to the following factors. Firstly, we utilize graph-based representations for both compounds and proteins, providing a more comprehensive and informative approach to encoding molecular structures compared to traditional sequence-based methods. By constructing three types of graphs, including drug molecule graphs, motif graphs, and protein graphs, our model captures the molecular structure and functional information from multiple perspectives, allowing for more accurate predictions of DTA. Secondly, during the feature representation learning stage, our model integrates multi-scale feature information using graph neural networks (GNNs). This enables the learning of enriched and informative molecular representations, leading to further improvements in predictive performance. Thirdly, the incorporation of attention mechanisms during the DTA prediction phase allows the model to adaptively fuse critical features, resulting in even higher accuracy. Importantly, experimental results demonstrate the potential of MSGNN-DTA for practical applications in drug discovery and development.

**Figure 1:**
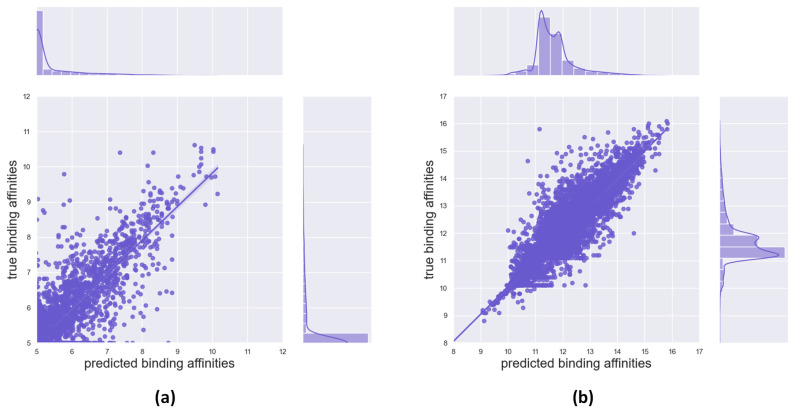
Scatter plot of true and predicted values on the Davis (**a**) and KIBA datasets (**b**), in which the horizontal coordinates represent the predicted binding affinity and the vertical coordinates represent the true binding affinity. The bar charts above and right show the distribution of the sample size.

Figure 1 displays the relationship between the predicted binding affinity and the true value. Upon analysing the model prediction, the linear regression curves between the true and predicted values are almost indistinguishable from the diagonal line, indicating an excellent fit between the predicted and true values. Moreover, the distribution trend of the sample size between the predicted and actual values closely aligns, further validating the model’s accuracy in making precise predictions.

### 2.4. Performance Comparison of Various GNN Models and Pooling Methods

To achieve effective feature extraction with rich information during GNN-based representation learning, selecting the appropriate GNN model and pooling method is crucial. In this study, we conducted an evaluation of two different graph convolution methods, namely GCN and GAT, along with two distinct pooling methods, max pooling and average pooling, to obtain the graph-level representations of drugs and proteins, as presented in Table 4. Our experimental findings reveal that GAT-based feature extraction outperforms GCN-based feature extraction in almost all performance metrics. This superiority can be attributed to the multi-head attention mechanism employed by GAT, which aggregates neighbouring node features and considers node correlation by computing attention scores, whereas GCN assigns the same attention weight to different neighbouring nodes. Furthermore, we observed that the max pooling method yields higher prediction accuracy than the average pooling method on both benchmark datasets. This finding highlights the importance of selecting a suitable pooling method in GNN-based models for DTA prediction.

### 2.5. Ablation Experiments

To investigate the key factors influencing the predictive performance of our model, we conducted a series of ablation experiments using the following variants of MSGNN-DAT:

- Without Attention: This model does not incorporate an attention mechanism to fuse the feature representations of the three channels, instead it directly concatenates the features to predict DTA, which is equivalent to giving the three parts equally important weight parameters.
- Without Motif-Level: This model does not construct a motif graph to learn drug motif-level feature representation, instead it only constructs a drug atom graph and a weight protein graph, and fuses two parts to predict DTA
- Without Skip-Connection: This model does not incorporate the gated skip-connection mechanism during the feature learning process, the node features of the previous-hop are not preserved when aggregating the next-hop neighbour information, and the hidden features at different scales are discarded.

Through these ablation experiments, we can gain insight into the relative importance of each component in our proposed model and the effectiveness of our design choices. Table 5 depicts the results of our ablation experiments on the two benchmark datasets, highlighting the superior performance of MSGNN-DTA over all other variants. Particularly noteworthy is the considerable performance gap between MSGNN-DTA and the other variants when the attention mechanism is not employed. This finding highlights the crucial role of attention in our model, as it permits the discerning integration of critical information during the feature aggregation process. Furthermore, we observe that the absence of motif-level results in comparatively poorer performance than MSGNN-DTA, emphasizing the importance of learning drug features from diverse perspectives and exploiting the topological information of drugs more comprehensively. Lastly, we note that the gated skip-connection mechanism can selectively preserve the features of different scales, resulting in further improvements in prediction performance.

### 2.6. Case Study

To evaluate the generalization capability of our model, we conducted experiments on a set of FDA-approved drug candidates from the DrugBank [33] database, excluding those contained in the KIBA dataset and retaining 2092 drug candidates. Subsequently, we selected a specific protein, epidermal growth factor receptor (EGFR), which is known to be associated with various types of cancer and is a popular target for cancer therapy. Among the 2092 candidates, 9 are known to have interactions with EGFR. We used the trained model on the KIBA dataset to calculate the interaction scores between all of the drug candidates with EGFR, ranked in descending order of scores for further analysis.

The results presented in Table 6 demonstrate that out of the top 11 small molecule compounds, 6 of them are EGFR inhibitors, while the remaining 3 compounds are ranked at positions 17, 32, and 43. Several other top-ranking drugs belong to tyrosine kinase inhibitors, which are targeted therapies for various types of cancer. Given that EGFR is a member of the tyrosine kinase family, these drugs possess a high possibility to be ligands for binding to EGFR. This assertion is supported by existing literature, where the mode of action of ibrutinib with mutant EGFR kinases has been investigated [34,35].

**Figure 2:**
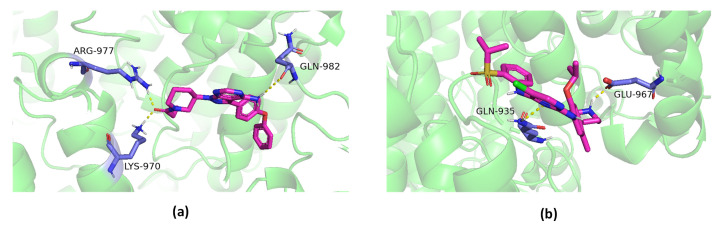
(**a**) Molecular docking and hydrogen bonding colouring results between 5YU9 and ibrutinib (DB09053). (**b**) Molecular docking and hydrogen bonding colouring results between 5YU9 and ceritinib (DB09063); the target protein is shown as a cartoon (green), the ligand molecule is shown as a stick structure (pink), the hydrogen bonding is shown in yellow, and the amino acid residues connected to the ligand by hydrogen bonding are shown as stick structures (purple).

To further validate the predicted drug–target interactions, we downloaded the crystal structure of EGFR (UniProt P00533) with PDB ID 5YU9 from the Protein Data Bank (PDB) and performed molecular docking using Autodock [36]. We used the lowest affinity energy output as a candidate binding site for specific ligands and receptors, thereby visualizing the hydrogen bonds formed by docking between drug molecules and amino acids of proteins using Pymol, as shown in Figure 2.

Our results demonstrate that the MSGNN-DTA exhibits a strong generalization performance in identifying potential drug candidates that have a high likelihood of binding to specific targets among a massive number of candidates. This makes it a valuable tool for screening potential drug candidates and prioritizing those with a higher predicted binding affinity for further testing. Ultimately, this could lead to the development of more effective drugs with improved therapeutic outcomes and fewer side effects.

## 3. Discussion

In this study, we introduce a novel approach for predicting drug–target binding affinity named MSGNN-DTA. Our method utilizes a graph neural network that introduces a gated skip-connection mechanism, which integrates multi-scale topological features to improve the accuracy of predictions. Specifically, we construct drug atom-level graphs, motif-level graphs, and weighted protein graphs to capture more sufficient information about drugs and proteins. Additionally, we incorporate an attention mechanism to adaptively fuse the multi-scale features, which enhances the performance of DTA prediction. The proposed method has the potential to significantly advance drug discovery and contribute to the development of more effective treatments.

The results demonstrate that our proposed method significantly outperforms the baseline approach. In practical applications, our pre-trained model can predict the affinity value by simply inputting the SMILES string of the drug and the amino acid sequence of the protein. This provides a powerful tool for the virtual screening of target proteins, facilitating the discovery of lead compounds. Although MSGNN-DTA shows superior performance in DTA prediction, there is still scope for further improvements.

The overall 3D geometry of a compound plays a crucial role in the interactions between drugs and protein targets. For instance, the active site of a protein often has specific geometric constraints, and the overall 3D shape of a drug must match it to effectively bind and exert its function. Furthermore, for compounds with multiple chiral centres, different optical isomers can have distinct biological activities, particularly for compounds such as protein kinase inhibitors. Thus, the correct selection and optimization of optical isomers are crucial in drug design and discovery. In this study, we did not consider the optical isomers and the overall 3D geometry of compounds, which may limit the predictive ability of our method. Although our method achieved good results in predicting based on molecular topological structure, its predictive ability may be limited for some complex compounds.

In future research, we will further explore how to incorporate optical isomers and the overall 3D geometry of compounds into our model. This will include using advanced computational tools to simulate the 3D shape of molecules and developing new models to process this information. We believe that this work will help improve the predictive performance of our method and apply it to a wider range of compounds and proteins.

## 4. Materials and Methods

### 4.1. Datasets

In this research, we performed a comprehensive performance evaluation of MSGNN-DTA on two widely recognized and publicly available datasets, namely the Davis [30] and KIBA [31] datasets. To ensure a fair and objective comparison, we employed a standard dataset split approach by randomly dividing the dataset into five parts, out of which four parts were used for training purposes while the remaining part was reserved for testing. We conducted five-fold cross-validation and reported the average performance as the final evaluation.

The Davis dataset contains 442 kinase proteins and their associated 68 inhibitors, with the binding affinity obtained through the measurement of dissociation constants $\left(K_{d}\right)$, which are expressed in units of nanomolar. To more graphically describe the relationship between $K_{d}$ and binding affinity, the $K_{d}$ was converted to logarithmic space with $pK_{d}$ [16], and the process of taking the negative logarithm is expressed in Equation (6). The higher value of $pK_{d}$ indicates a stronger binding affinity, with values ranging from 5.0 to 10.8. The boundary value 5.0 is considered the true negative drug–target pairs that exhibit either extremely weak binding affinities or are not detected in wet laboratory experiments.

(6) $$pK_{d}=-\log_{10}\left(\frac{K_{d}}{10^{9}}\right)$$

The KIBA dataset is a comprehensive and expansive resource. The interaction value was recorded using the KIBA score, derived from the combination of heterogeneous information sources, including the inhibition constant ($K_{i}$), $K_{d}$, and the half-maximal inhibitory concentration ($IC_{50}$), with values ranging from 0.0 to 17.2. The dataset is of superior quality, as the integrated heterogeneous measurements mitigated the data inconsistency arising from relying on a single information source. For further clarity, Table 7 presents a comprehensive overview of both benchmark datasets.

### 4.2. Model Architecture

**Figure 3:**
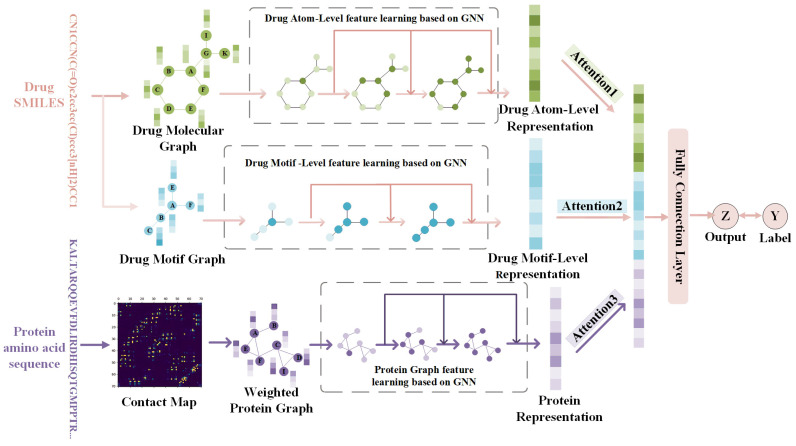
The main architecture of MSGNN-DTA.

Our prediction task aims to predict the binding affinity between drug–target pairs, given the SMILES of drugs and the amino acid sequence of target proteins as the original input. To achieve this goal, we propose a new approach called MSGNN-DTA, which involves constructing drug and protein graphs from multiple perspectives. For each drug, we simultaneously construct an atom graph and a motif graph, where individual atoms and motifs are represented as nodes, respectively. Meanwhile, for each protein, we predict residue contact maps using a protein structure prediction model and construct a weighted protein graph accordingly. To obtain multi-scale topological feature representations of drugs and proteins, we parallelize the constructed graphs through a GNN-based feature learning module. This module enables us to obtain two representations of the drug and one representation of the protein. Subsequently, we apply an attention mechanism to adaptively fuse the drug–target representations and obtain a joint representation, which is then fed into multiple fully connected layers to predict DTA. The main architecture of our model is illustrated in Figure 3, which depicts the detailed workflow of each module. In the following sections, we will provide a comprehensive description of each module.

### 4.3. Graph Construction for Drugs and Proteins

#### 4.3.1. Construction of Drug Atom-Level Graph

Drugs are commonly represented by SMILES (simplified molecular input line entry specification) [37]. The structural information of the molecule is missing when using the string representation directly. Therefore, we use the open-source molecular processing software Rdkit [38] to construct the atom-level graph of drugs based on SMILES, where nodes represent atoms, edges represent chemical bonds, and the graph topology is represented by an adjacency matrix A. The initial feature vector of each atom is obtained based on chemical and structural properties. The detailed meaning of node features is illustrated in Table 8, represented by a 78-dimensional vector.

#### 4.3.2. Construction of Drug Motif-Level Graph

**Figure 4:**
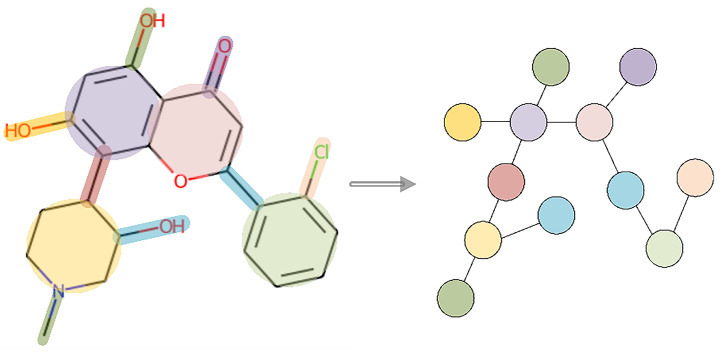
The construction process of a drug motif-level graph, where the nodes represent motifs and edges represent whether two nodes are connected by a chemical bond.

It is widely recognized that some motifs in drugs, such as the benzene ring, are intimately related to molecular properties. The benzene ring is meaningful when considered as a whole, but it loses meaning when the chemical bonds within the ring are separated individually. However, several layers of GNN cannot capture all of the information in the ring to which an atom belongs, resulting in incomplete information being extracted. Therefore, in MSGGN-DTA, the motif-level graph for drugs is constructed simultaneously. Cyclic structures and individual chemical bonds, which do not belong to any cyclic structures, along with their connected pairs of atoms, are considered the fundamental building blocks of molecules and are represented as nodes in the molecular motif graph [39]. Specifically, cyclic structure nodes represent a group of atoms and chemical bonds connected cyclically, while nodes representing individual chemical bonds along with their connected pairs of atoms represent the relationships between atoms and chemical bonds. This approach provides a better reflection of the structural information of the molecule, thus facilitating motif graph generation and model training. The edges represent whether two nodes are connected by a chemical bond. The construction process of a drug motif graph is depicted in Figure 4. Similar to the molecular graph, the initial features of nodes also need to be extracted. The detailed meaning is described in Table 9, represented by a 92-dimensional vector.

#### 4.3.3. Construction of Weighted Protein Graph

Proteins are conventionally represented as 1D sequences consisting of 25 distinct amino acids, but such a representation fails to reflect the entire spatial structure information. The spatial structure of proteins is determined by various interactions, including hydrogen bonds, ionic bonds, and hydrophobic interactions, among others [40]. Consequently, sole reliance on a 1D representation proves inadequate to capture the intricate spatial structure information of proteins, thereby posing a challenge in extracting an effective protein representation. Despite the exponential growth of protein databases, the structures of the majority of proteins remain unknown. However, recent advances in natural language processing techniques have facilitated the development of several cutting-edge protein language models [41,42,43], which can accurately predict protein structures solely from the input protein sequences.

In this study, we employed the ESM-1b model proposed by Rives et al. [42] to predict the contact map of proteins. The ESM-1b model is an unsupervised protein language modelling approach based on transformer architecture that leverages large-scale protein sequence and structure exploration through pre-training. It can accurately and efficiently predict protein contact maps by directly inputting the 1D protein sequence. We selected this model because ESM-1b can predict protein contact maps accurately without requiring multiple sequence alignment (MSA), which greatly enhances the prediction efficiency.

The contact map predicted by the ESM-1b model is represented as a probability matrix, where each element represents the interaction probability between different residues, ranging from 0 to 1. According to the construction process of the weighted protein graph in the WGNN-DTA model [25], if a value in the probability matrix exceeds the threshold of 0.5 is retained, while those below are set to 0. The weighted protein graph is constructed using residues as nodes, residue interactions as edges, and probability values as the weight of the edges. Since the ESM-1b model is trained with a fixed context size of 1024 tokens for position embedding, the sequence length is limited. To handle longer protein sequences (over 1000 residues), WGNN-DTA employs a truncation and splicing strategy to construct the contact map. The entire sequence is divided into multiple fixed-length subsequences of length *L* with a step size of L/2, and the contact map of each subsequence is predicted sequentially by the ESM-1b model, followed by splicing together, with overlapping parts averaged. Algorithm 1 describes the specific construction process of the contact map. Additionally, features of each residue node, such as residue type, polarity, hydrophobicity, weight, group dissociation constant, and more, are extracted to generate an initial feature vector for each node, represented by a 33-dimensional vector.
**Algorithm 1** Construction of a protein contact map**Input:** protein amino acid sequence: seq
**Output:** contact map
**Initialization:** contact map ←zeros(Len(seq),Len(seq)),

$\;window_{size}\leftarrow 500$
1:**if** Len(seq)<=1000 **then**2:      contact map ← ESM-1b (seq)3:**else**4:    $L\leftarrow len\left(seq\right)/window_{size}$5:    **for** i=0→L−2 **do**6:        $start\leftarrow i*window_{size}$7:        $end\leftarrow min\left\{\left(i+2\right)*window_{size},len\left(seq\right)\right\}$8:        subsequences ←seq[start,end]9:        temp contact map ← ESM-1b (subsequences)10:      row,col← The non-zero rows and columns in the contact map [start,end]11:      row←row+start, col←col+start12:      contact map [start,end]← contact map [start,end] + temp contact map13:      contact map [row,col]← contact map [row,col]/214:    **end for**15:**end if**
**Return:** contact map


### 4.4. Feature Learning Based on Graph Neural Networks

Through the process of graph construction, we obtain the drug atom-level graph, the motif-level graph, and the weighted protein graph. GNN can effectively extract hidden features using the spatial topological structure information of the graph, and obtain a graph-level representation by aggregating features of nodes. Below is a brief description of the graph convolutional network (GCN) [44] and the graph attention network (GAT) [45].

For a graph G=(V,E), V is the set of nodes and E is the set of edges. The initial feature vector of each atom is $X_{i}$, a graph is represented by a feature matrix $X\in R^{N*F}$ and an adjacency matrix $A\in R^{N*N}$, where *N* is the number of nodes, *F* is the feature dimension, and the adjacency matrix represents the interaction relationship between nodes. The propagation mechanism of the GCN layer is described in Equation (7).

(7) $$H^{\left(l+1\right)}=\sigma \left({\tilde{D}}^{-\frac{1}{2}}\tilde{A}{\tilde{D}}^{-\frac{1}{2}}H^{\left(l\right)}W^{\left(l\right)}\right)$$

where $\tilde{A}$ is the adjacency matrix added to a self-loop, $\tilde{D}$ is the degree matrix of the graph, and $H^{\left(l\right)}$ represents the feature matrix of the *l*th layer. $H^{\left(l+1\right)}$ represents the output of the feature representation after message propagation. σ is the ReLU activation function. *W* is a learnable weight matrix. The input layer $H^{\left(0\right)}$ is equal to the input feature matrix *X*.

GAT learns the hidden representation of nodes based on the self-attentive mechanism. First, the nodes are linearly transformed by a weight matrix $W\in R^{F^{\prime }*F}$, and $F^{\prime }$ denotes the feature dimension of hidden layer nodes. For a given node *i*, the attention coefficient with its neighbour *j* is calculated by Equations (8) and (9). The attention weights are then normalized with their neighbouring nodes using the Softmax function to ensure that the sum of attention weights of all neighbouring nodes is equal to one, indicating the importance between node pairs. The LeakyReLU activation function is used to improve the model’s stability and robustness, especially when processing negative inputs, outperforming the ReLU activation function [45]. Equation (10) aggregates the features of neighbouring nodes according to the attention score to obtain the feature representation of the hidden layer.

(8) $$e_{ij}=a\left(WX_{i}∥WX_{j}\right)$$

(9) $$\alpha_{ij}=\mathrm{softmax}\left(e_{ij}\right)=\frac{\exp\left(\mathrm{LeakyReLU}\left(e_{ij}\right)\right)}{\sum_{k\in N_{i}}\exp\left(\mathrm{LeakyReLU}\left(e_{ik}\right)\right)}$$

(10) $$h_{i}=\sigma \left(\sum_{j\in N_{i}}\alpha_{ij}WX_{j}\right)$$

where $X_{i}$ is feature vector of node *i*, $N_{i}$ is the set of neighbouring nodes of node *i*, $e_{ij}$ denotes the attention coefficient between node *i* and node *j*, $\alpha_{ij}$ denotes the normalized attention coefficient, $h_{i}$ is the hidden layer feature of node *i*, σ is the non-linear activation function, and *a*, *W* is the learnable weight matrix.

In MSGNN-DTA, node-level feature representations $z\in R^{N*F}$ are learned through three consecutive GNN layers. To obtain representation vectors of the same length for drugs containing different atom numbers and proteins with different residue numbers, we add pooling layers after the last GNN layer, aggregating node-level features to obtain graph-level representations. Finally, a 128-dimensional vector is obtained by several fully connected and dropout layers.

#### Gated Skip-Connection Mechanism

To aggregate neighbour information at long distances in a specific central atom, stacking multiple GNN layers is necessary. However, the side effects of gradient disappearance and node degradation appear as the number of GNN layers increase. We incorporate a gated skip-connection mechanism [46] in the representation learning of each hidden layer, fusing features from different hidden states by adjusting the rate of forgetting and updating. Along with the increase in model depth, each node can aggregate the information carried by remote nodes and preserve the unique features of the node themselves.

The gated skip-connection mechanism is described in Equations (11) and (12).

(11) $$z_{i}=\mathrm{sigmoid}\left(U_{1}H_{i}^{\left(l+1\right)}+U_{2}H_{i}^{\left(l\right)}+b\right)$$

(12) $$H_{i}^{\left(l+1\right)}=z_{i}H_{i}^{\left(l+1\right)}+\left(1-z_{i}\right)H_{i}^{\left(l\right)}$$

where $U_{1}$ and $U_{2}$ are trainable parameters, *b* is bias, $H_{i}^{\left(l\right)}$ and $H_{i}^{\left(l+1\right)}$ denote the *l*th and *l*+1th layer feature vectors of node *i*, respectively, and $z_{i}$ is the learned proportion coefficient that retains the information of the previous hidden layer. Here we have chosen a sigmoid activation function to ensure that the learned proportion coefficient falls within the range of 0 to 1.

### 4.5. Prediction of Drug–Target Binding Affinity

With three representation learning modules running in parallel, we obtain drug atom-level ($Z_{d}$), drug motif-level ($Z_{m}$), and protein ($Z_{p}$) representations. The three parts are concatenated into a complete vector and then fed into three consecutive fully connected layers to predict DTA.

Compared with many previous models that employ simple concatenation, the attention mechanism allows the model to adaptively integrate the critical features, further improving prediction accuracy. Let $\alpha_{d}$, $\alpha_{m}$, and $\alpha_{p}$ denote the attention scores of $Z_{d}$, $Z_{m}$, and $Z_{p}$, respectively. Firstly, the weight scores $w_{d}$, $w_{m}$, and $w_{p}$ are calculated by Equation (13). Here we choose the tanh activation function, which increases the speed of model convergence.

(13) $$w_{i}=W_{2}\tanh\left(W_{1}Z_{i}\right)\;i=d,m,p$$

where $W_{1}$ and $W_{2}$ are learnable weight vectors and can be adjusted during training, and then normalized using by the Softmax function to map the above-learned weight scores to the (0,1) interval to obtain the attention scores, which represent the importance of each part in determining the final prediction.

(14) $$\alpha_{i}=\mathrm{softmax}\left(w_{i}\right)=\frac{e^{w_{i}}}{e^{w_{d}}+e^{w_{m}}+e^{w_{p}}}$$

Finally, we connect the three components of the representation by the learning attention scores.

(15) $$Z_{c}=\alpha_{d}Z_{d}∥\alpha_{m}Z_{m}∥\alpha_{p}Z_{p}$$

where $Z_{c}$ denotes the connected feature vector of the drug–target pair.

## 5. Conclusions

The study proposes a novel approach, MSGNN-DTA, for predicting drug–target binding affinity that integrates multi-scale topological features using graph neural networks. We concurrently construct drug atom-level graphs, motif-level graphs, and weighted protein graphs for learning enhanced multi-scale features that represent the rich information of drugs and proteins. The novelty of this approach lies in its ability to capture the multi-scale topological features of drugs and proteins and fuse them adaptively using an attention mechanism. This allows for more a accurate prediction of the drug–target binding affinity and has the potential to aid in the development of more effective and safe drugs with fewer adverse effects.

The proposed method is evaluated through a series of experiments, which demonstrate it outperforms existing state-of-the-art models in all evaluation metrics, indicating its potential as a powerful tool for accurate DTA prediction. Furthermore, we conducted an analysis of candidate drugs for the epidermal growth factor receptor (EGFR) based on FDA-approved drugs, and the predicted scores of drugs known to interact with EGFR were consistently ranked among the top positions, further validating the effectiveness and generalization ability of the proposed method. These results collectively highlight the potential of MSGNN-DTA as an efficient and reliable approach for advancing drug discovery and design. These results collectively highlight the potential of MSGNN-DTA as an efficient and reliable approach for advancing drug discovery and design.

In future work, we plan to investigate the feature representation learning process further by integrating a broader range of features, including evolutionary, structural, functional, and physicochemical features, among others. Additionally, we will explore the construction of networks using similarity matrices to enhance the accuracy of DTA prediction. Our research directions aim to continue advancing the field of drug–target interaction prediction and contribute to the development of more effective therapeutic interventions.

## Figures and Tables

**Table 1:** Experimental hyperparameter settings.

Parameters	Setting
Epoch	2000
Batch size	512
Leaning rate	0.0005
Optimizer	Adam
Dropout rate	0.2
Graph convolutional layers	3
Input dimension of the three layers in GNN	N, 4 N, 4 N
Output dimension of the three layers in GNN	N, 4 N, 4 N
Fully connected layer hidden unit	1024, 512

Note: N represents the dimension of the initial features.

**Table 2:** Performance evaluation of the DTA prediction models on the Davis dataset.

Methods	Proteins	Compounds	MSE ↓	CI ↑	$r_{m}^{2}$↑	Pearson ↑
KronRLS	Smith–Waterman	Pubchem-Sim	0.379	0.871	0.407	-
SimBoost	Smith–Waterman	Pubchem-Sim	0.282	0.872	0.644	-
DeepDTA	CNN	CNN	0.261	0.878	0.630	-
WideDTA	CNN + PDM	CNN + LMCS	0.262	0.886	-	0.820
GraphDTA	CNN	GIN	0.229	0.893	-	-
GEFA	GCN	GCN	0.228	0.893	-	0.847
MGraphDTA	MCNN	MGNN	0.207	0.900	0.710	-
WGNN-DTA	GCN	GCN	0.208	0.900	0.692	0.861
WGNN-DTA	GAT	GAT	0.208	0.903	0.691	0.863
DGraphDTA	GCN	GCN	0.202	0.904	0.700	0.867
MSGNN-DTA	GAT	GAT + GAT	**0.195**	**0.906**	**0.719**	**0.871**

Note: Bold indicates the best result in the evaluation metrics. These results are not reported from original studies.

**Table 3:** Performance evaluation of the DTA prediction models on the KIBA dataset.

Methods	Proteins	Compounds	MSE ↓	CI ↑	$r_{m}^{2}$↑	Pearson ↑
KronRLS	Smith–Waterman	Pubchem-Sim	0.411	0.782	0.342	-
SimBoost	Smith–Waterman	Pubchem-Sim	0.222	0.836	0.629	-
DeepDTA	CNN	CNN	0.194	0.863	0.673	-
WideDTA	CNN + PDM	CNN + LMCS	0.179	0.875	-	0.856
GraphDTA	CNN	GAT − GCN	0.139	0.891	-	-
MGraphDTA	MCNN	MGNN	0.128	0.902	0.801	-
WGNN-DTA	GCN	GCN	0.144	0.885	0.781	0.888
WGNN-DTA	GAT	GAT	0.130	0.898	0.791	0.899
DGraphDTA	GCN	GCN	0.126	0.904	0.786	0.903
MSGNN-DTA	GAT	GAT + GAT	**0.117**	**0.908**	**0.818**	**0.910**

Note: Bold indicates the best result in the evaluation metrics. These results are not reported from original studies.

**Table 4:** Performance comparison of different GNN models and pooling methods.

Dataset	GNN Model	Pooling Method	MSE ↓	CI ↑	$r_{m}^{2}$↑	Pearson ↑
Davis	GCN	Max	0.203	0.903	0.713	0.864
	GCN	Mean	0.201	0.904	**0.723**	0.866
	GAT	Max	**0.196**	**0.906**	0.719	**0.871**
	GAT	Mean	0.202	0.905	0.716	0.865
KIBA	GCN	Max	0.122	0.904	0.789	0.906
	GCN	Mean	0.121	0.904	0.795	0.907
	GAT	Max	**0.117**	**0.908**	**0.818**	**0.910**
	GAT	Mean	0.122	0.906	0.794	0.906

Note: Bold indicates the best result of the evaluation metrics.

**Table 5:** Performance comparison of ablation experiments.

Dataset	Variants	MSE ↓	CI ↑	$r_{m}^{2}$↑	Pearson ↑
Davis	Without Motif-Level	0.200	0.903	0.715	0.867
	Without Skip-Connection	0.201	0.903	**0.728**	0.866
	Without Attension	0.203	0.897	0.709	0.865
	MSGNN-DTA	**0.195**	**0.906**	0.719	**0.871**
KIBA	Without Motif-Level	0.123	0.905	0.790	0.906
	Without Skip-Connection	0.122	0.904	0.788	0.906
	Without Attension	0.124	0.906	0.808	0.905
	MSGNN-DTA	**0.117**	**0.908**	**0.818**	**0.910**

Note: Bold indicates the best result of the evaluation metrics.

**Table 6:** The predicted KIBA score ranking of drug candidates with EGFR.

Rank	DrugBank ID	Drug Name	Predict KIBA Score
1	DB09053	Ibrutinib	12.98426
2	**DB00317**	**Gefitinib**	12.94488
3	**DB12267**	**Brigatinib**	12.91385
4	DB09063	Ceritinib	12.86993
5	DB12095	Telotristat ethyl	12.86020
6	DB01254	Dasatinib	12.71807
7	**DB01259**	**Lapatinib**	12.66030
8	**DB05294**	**Vandetanib**	12.61738
9	**DB11828**	**Neratinib**	12.61046
10	DB01167	Itraconazole	12.60930
11	**DB00530**	**Erlotinib**	12.58477

Note: Bold in the table denotes a drug that has known interactions with EGFR.

**Table 7:** Summary of the Davis and KIBA datasets.

Dataset	Compounds	Proteins	Interactions
Davis	68	442	30,056
KIBA	2111	229	118,254

**Table 8:** The node features for a drug atom-level graph.

Feature Name	Dimension
Atomic symbol	44
Degree of atom	11
Total number of connected hydrogen atoms (implicit and explicit)	11
Implicit valence of atoms	11
Whether the atom is aromatic or not	1

**Table 9:** The node features for a drug motif-level graph.

Feature Name	Dimension
Atomic symbols contained in the motif	44
Number of atoms in the motif	11
Number of edges connecting to other motifs	11
Total number of hydrogen atoms connected by motif (implicit & explicit)	12
Implicit valence of motif	12
Whether the motif is a simple ring	1
Whether the motif is chemically bonded or not	1

## Data Availability

The source code and data of this study are available at https://github.com/songxuanmo/MSGNN-DTA (accessed on 1 May 2023).

## Abbreviations

The following abbreviations are used in this manuscript:

DTA	Drug–Target Binding Affinity
GNNs	Graph Neural Networks
CNN	Convolutional Neural Networks
GCN	Graph Convolutional Network
GAT	Graph Attention Network
SMILES	Simplified Molecular Input Line Entry Specification
MSA	Multiple Sequence Alignment
MSE	Mean Squared Error
CI	Concordance Index
$r_{m}^{2}$	Regression Towards the Mean
$K_{d}$	Dissociation Constants
$K_{i}$	Inhibition Constant
$IC_{50}$	Half-Maximal Inhibitory Concentration
PYG	PyTorch Geometric
EGFR	Epidermal Growth Factor Receptor
